# Facile Synthesis of Polyaniline Nanotubes Using Self-Assembly Method Based on the Hydrogen Bonding: Mechanism and Application in Gas Sensing

**DOI:** 10.3390/polym9100544

**Published:** 2017-10-24

**Authors:** Changqing Yin, Lei Gao, Fei Zhou, Guotao Duan

**Affiliations:** 1 Key Lab of Materials Physics, Anhui Key Lab of Nanomaterials and Nanotechnology, Institute of Solid State Physics, Chinese Academy of Sciences, Hefei 230031, China; ycqdomain@163.com (C.Y.); lgao@issp.ac.cn (L.G.); fzhou@issp.ac.cn (F.Z.); 2 School of Chemistry and Materials Science, University of Science and Technology of China, Hefei 230026, China

**Keywords:** polyaniline nanotubes, self-assembly, gas sensing, high performance

## Abstract

Based on hydrogen bonding, the highly uniform polyaniline (PANI) nanotubes were synthesized by self-assembly method using citric acid (CA) as the dopant and the structure-directing agent by optimizing the molar ratio of CA to aniline monomer (Ani). Synthesis conditions like reaction temperature and mechanical stirring were considered to explore the effects of hydrogen bonding on the morphologies. The effects of CA on the final morphology of the products were also investigated. The as-synthesized CA doped polyaniline (PANI) nanomaterials were further deposited on the plate electrodes for the test of gas sensing performance to ammonia (NH_3_). The sensitivity to various concentrations of NH_3_, the repeatability, and the stability of the sensors were also tested and analyzed. As a result, it was found that the PANI nanomaterial synthesized at the CA/Ani molar ratio of 0.5 has highly uniform tubular morphology and shows the best sensing performance to NH_3_. It makes the PANI nanotubes a promising material for high performance gas sensing to NH_3_.

## 1. Introduction

Nowadays, environmental problems become more and more serious with the development of the society. Toxic and rotten gases, such as ammonia (NH_3_), widely exist in people’s daily life and seriously impact on people’s health. It has become a hot issue to realize fast detection and real-time monitoring of these toxic and rotten gases. Among all of the detection methods, semiconductor gas sensors, which use the semiconductor materials as the sensitive materials, have attracted much attention because of its high sensitivity, fast response time, small physical size, simple fabrication, and low cost [1,2]. Today, the vast used sensing materials are the metal-oxide semiconductor (MOS), such as SnO_2_, WO_3_, ZnO_2_, and Fe_2_O_3_ [3,4,5]. These MOS gas sensors processed remarkably high sensitivity and a large detection range to a variety of gases and were expected to be employed in domestic, industries and military applications [6]. However, MOS gas sensors generally only work at high temperature, and consequently results in high energy consumption and restriction in some special areas, for example, places with inflammable or explosive gases. So, it is quite urgent to explore room temperature sensing materials for the special working environment and with low energy consumption [7].

In the past decades, conducting polymers (CPs) have been extensively investigated in gas sensors as the room temperature sensing materials [8,9,10,11] due to its special sensing mechanism [12,13,14,15]. Among the various conducting polymers, polyaniline (PANI) is considered very important, due to its large-scale synthesis compatibility, low cost, good environmental stability, and tunable electrical properties [16,17,18,19]. Furthermore, it is believed that PANI at the nanoscale with special morphology shows additional preponderance for the increasing of surface-to-volume area and improving of surface active sites, which will further improve the sensing performance [20]. So it is very important to optimize the synthesis conditions and processes to fabricate PANI with specific morphologies and sizes of bulk quantities for high performance gas sensing. In many synthesis methods, hard-template method requires unique templates thay are often difficult to fabricate for the specific morphologies, and the following steps to remove the hard templates are complex, and possibly destroy the as-fabricated nanostructures [21,22]. Thus, it is limited in the fabrication of PANI nanostructures. On the other hand, the soft-template method using organic acids assisted as dopant and structure-directing agent through the self-assembly process has been widely investigated [23,24,25,26,27,28]. For example, PANI nanofibers synthesized by interfacial polymerization and self-assembly had optimized sensing performances because of the high doping level of hydrogen chloride (HCl) [28]. PANI nanotubes with special morphology and void structure will provide more surface-to-volume area and surface active sites, which will improve the sensing performances. At the same time, the ratio and kind of doped organic acids has a significant impact on the morphology of the PANI, which is also closely related to the properties of gas sensors.

Base on this, uniform PANI nanotubes were investigated via using an in-situ soft-template chemical method in the presence of citric acid (CA), which served both as dopant and structure-directing agent by the self-assembly method. The morphologies of PANI nanostructures were closely related to the molar ratio of CA to aniline (Ani) monomer, noted as CA/Ani. With the increase of CA/Ani ratio, PANI nanostructures undergo the transformation from nanoflowers to nanotubes and nanobranches. In order to prove the effect of the hydrogen bonding, reaction temperature and mechanical stirring were also set as variables to regulate the morphology. The formation mechanism and the gas sensing properties to ammonia (NH_3_) will be discussed in this paper later. The gas sensing performances indicate that the PANI nanotubes exhibit good response and stability properties to ammonia (NH_3_), which may provide a new approach in gas sensing.

## 2. Experimental

### 2.1. Materials

Aniline (C_6_H_7_N, Ani), methanol, ethanol, Potassium bromide (KBr), Ammonium persulfate ((NH_4_)_2_S_2_O_8_, APS), and citric acid (CA) were all purchased from Sinopharm Chemical Reagent limited corporation, Shanghai, China. All of the chemical reagents were all analytical reagent (AR) and used directly without any further purification. Deionized water (DI water) was obtained from an ultrapure water system (Milli-Q, Millipore, MA, Molsheim, France). All of the solutions were prepared in DI water.

### 2.2. Synthesis of PANI Nanotubes

In a typical synthesis process of PANI nanotubes is described as follows: 1 mmol citric acid (CA) was dissolved in 10 mL DI water with sonication treatment for 30 min to get the homogeneous solution. Then, 2 mmol of Ani was added into the CA/water solution and stirred for 30 min, and the pale yellow solution was then stored in ice bath (low-temperature thermostat bath, with the temperature control accuracy less than 0.5 °C), and the pH value of the monomer system is 4. Next, the well-cooled 5 mL of 0.4 mmol/mL APS aqueous solution was added drop by drop into the Ani/CA/water solution, and the pH value of the reaction system can reach to 1. The resultant solution was allowed to stand for 24 h in the ice bath. Finally, the resulting precipitate was collected and washed several times with DI water, ethanol and methanol, respectively. The obtained products were then dried in a vacuum oven at 50 °C for 24 h for further use.

To explore the influence of the amount of CA on the morphologies of the final products, the molar ratio of CA to Ani, noted as CA/Ani, was changed from 0.01 to 2 with the certain amount of Ani, and the experiments were carried out in ice bath for 24 h. The influence of the amount of APS was also considered, the experiments were executed with CA/Ani = 0.5 and in ice bath for 24 h, and the molar ratios of APS to Ani, noted as APS/Ani, was ranged from 0.25 to 2. Then, the effects of temperature and stirring were also discussed to optimize the forming conditions of the PANI nanotubes with CA/Ani = 0.5 and APS/Ani = 1 by making the reactions in room temperature or with stirring.

### 2.3. Characterization Methods

The morphologies of the as-synthesized products were observed by field-emission scanning electron microscopy (SEM, Hitachi, SU8020, Tokyo, Japan) and the high-resolution transmission electron microscopy (TEM, JOEL, JEM-2010, Tokyo, Japan). FTIR spectra of the samples were performed by a NICOLET MX-1E Fourier transformed spectrometer with KBr tablet (Thermo Fisher Scientific, Waltham, MA, USA). Raman spectra of the samples were carried out by a Nexus Infrared—Laser Raman spectrometer (Renishaw, London, UK) with the samples deposited on the glass slides. X-ray diffraction (XRD) was measured on an X-ray diffractometer (Philips X’Pert, Almelo, The Netherlands) with a Cu-K a line (0.15419 nm). The statistics of the size of the products were carried out by importing the SEM images into the nanaomeasurer (1.2) software (Hitachi Limited, Tokyo, Japan) and selecting 30 dots randomly for calculation.

### 2.4. Sensors Fabrication and the Gas Sensing Measurements

The as synthesized products were dispersed in the mixture of DI water and ethanol with the proportion of 1:1 with ultrasonic treatment for 30 min. Then, 2 μL of the dispersion was dropped on a plate electrode, which was insulated between the two electrodes and the distance between the two electrodes was about 200 μm. The as-fabricated sensor was dried out in air ambient and welded onto a sensor base for the further sensing tests.

The gas sensing performances of the sensors were tested in a static system. In particular, the sensors and a fixed resistor with the values ranging from 0.1 to 100 Mohm were connected in series on a circuit board. A DC power supply (Agilent U8002A, San Jose, CA, USA) provided a 10 V regulated power supply, and the voltage change on the fixed resistor was collected by a multimeter (Agilent mod. U3606A) and recorded by the computer. The testing ambience was made by injecting the target gas with a certain amount, which was calculated by the ideal gas equation of state. All of the tests were carried out in air ambient at room temperature (25 °C).

## 3. Results and Discussion

### 3.1. Structure Characterization of PANI Nanotubes

**Figure 1:**
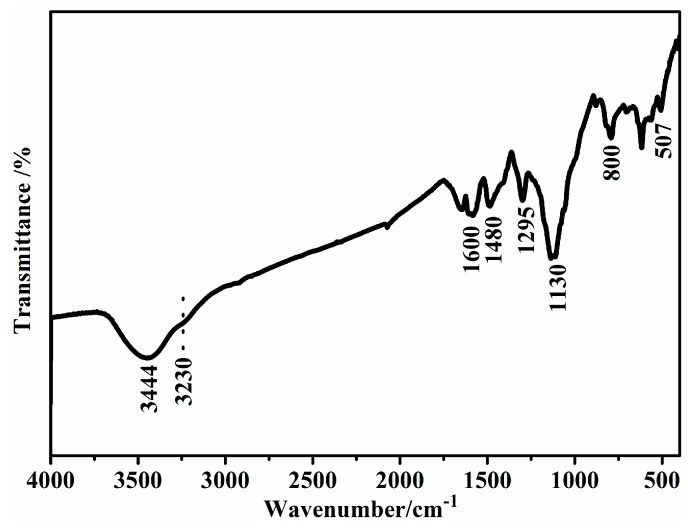
FTIR spectrum of the product prepared at CA/Ani = 0.5, APS/Ani = 1 with Ani of 2 mmol and ice bath for 24 h.

In order to demonstrate the structure and formation mechanism of the products, fourier transform infrared spectroscopy (FTIR) spectrum of the sample was measured. The FTIR spectrum of the product prepared at CA/Ani = 0.5, APS/Ani = 1 with Ani of 2 mmol and ice bath for 24 h was shown in Figure 1. As can be seen in the spectrum, the main adsorption peaks are in good agreement with the PANI structure in the previous reports [29]. In details, the peaks around 1584 and 1500 cm^−1^ were from the C=C stretching vibration of quiniod and benzenoid ring [27], respectively. The two absorptions indicated that the PANI has the amine and imine nitrogen unites in its polymer chains [25,30]. The CN stretching vibration [31] centered at about 1300 cm^−1^. The adsorption at about 821 cm^−1^ was ascribed to the CH out-of-plane bending in the 1,4-disubstituded benzene ring [32,33]. In particular, the stretching vibration of CH_2_ group at about 2930 and 2855 cm^−1^, and the absorption at 1046 and 509 cm^−1^, which came from the COOH group stretching vibration were observed, indicating the as-prepared PANI were doped with citric acid [34,35]. On the other hand, the broad absorption at 3444 cm^−1^ was due to the OH stretching vibration [36].

**Figure 2:**
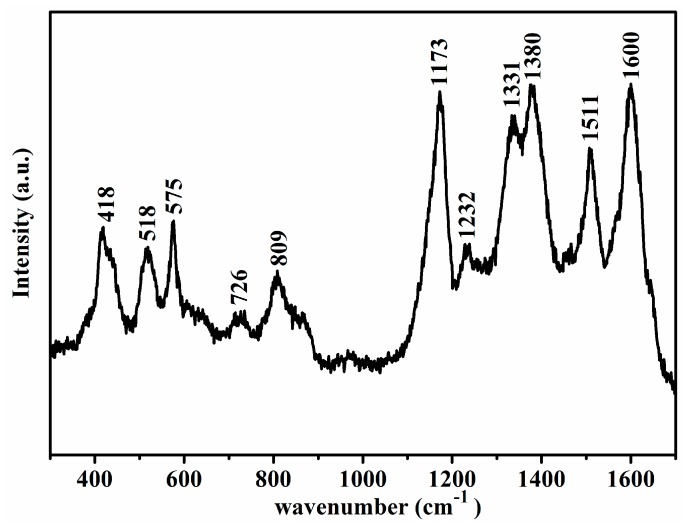
Raman spectrum of the product prepared at CA/Ani = 0.5, APS/Ani = 1 with Ani of 2 mmol and ice bath for 24 h.

To further investigate the molecular information in conjunction with FTIR spectrum, Raman scattering of the above PANI sample was measured as shown in Figure 2. The band at 1174 and 1597 cm^−1^ were due to C–H bending of the benzenoid ring and C=C stretching of the quinoid ring [37], respectively. The absorption at about 1239 cm^−1^ came from C–N stretching mode of the single bond [38], and the peak at 1456 cm^−1^ refer to C=N stretching mode of the quinoid units [39], which indicated the presence of doped PANI structure. Furthermore, the bands at 1343, 1390, and 1508 cm^−1^ were from the C–N^+^ stretching of the bipolaron structure, the C=C stretching vibration of the quinoid, and the N–H bending of the bipolaronic structure [21,40,41]. The peaks at 572 and 607 cm^−1^ were also observed, which were the amine deformation and benzenoid ring deformation [42]. The peak at 417 and 811 cm^−1^ were attributed to the C–H deformation, and the peak at 530 cm^−1^ was due to the C=N–C torsion [43]. The above discussions reveal that the as-synthesized PANI was in a doped state, and this result was also proved by the XRD analysis.

### 3.2. Morphology and the Growth Mechanism

**Figure 3:**
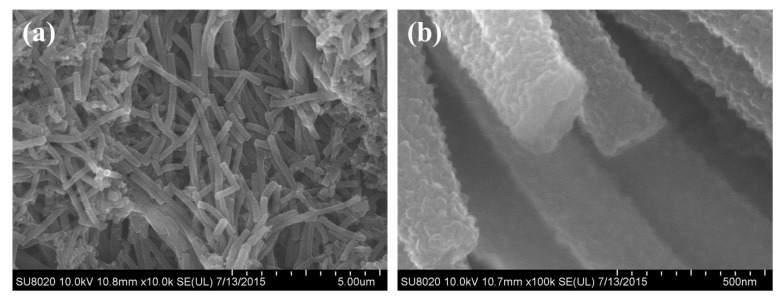
Scanning electron microscopy (SEM) images of the polyaniline (PANI) nanotubes prepared at CA/Ani = 0.5, APS/Ani = 1 with Ani of 2 mmol and ice bath for 24 h. (**a**) the SEM image of PANI with a scale of 5 μm; (**b**) the SEM image with a scale of 500 nm.

**Figure 4:**
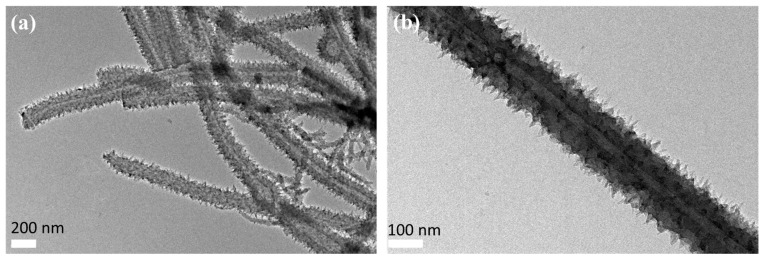
Transmission electron microscopy (TEM) images of the PANI nanotubes prepared at CA/Ani = 0.5, APS/Ani = 1 with Ani of 2 mmol and ice bath for 24 h. (**a**) the TEM image of PANI with a scale of 200 nm; (**b**) the TEM image with a scale of 100 nm.

For the as synthesized PANI sample, the SEM and TEM characterizations were carried out and the results were shown in Figure 3 and Figure 4. From the SEM images of Figure 3a, we can see that lots of uniform nanotubes were obtained. The diameter of the nanotubes ranged from 180 to 230 nm, and the average diameter was about 210 nm. From the statistical analysis of the particle size, we can also find that the as formed PANI nanotubes were evenly distributed. From the SEM image we can also see that the surface of PANI nanotubes was quite scratchy, which can be further proved by the TEM observation. The tubular structure was proved by high resolution SEM imagery, as shown in Figure 3b and the TEM observation. So we can preliminarily concluded high quantity uniform PANI nanotubes can be obtained when using CA as dopant and structure directing agent in a specific reaction conditions.

**Figure 5:**
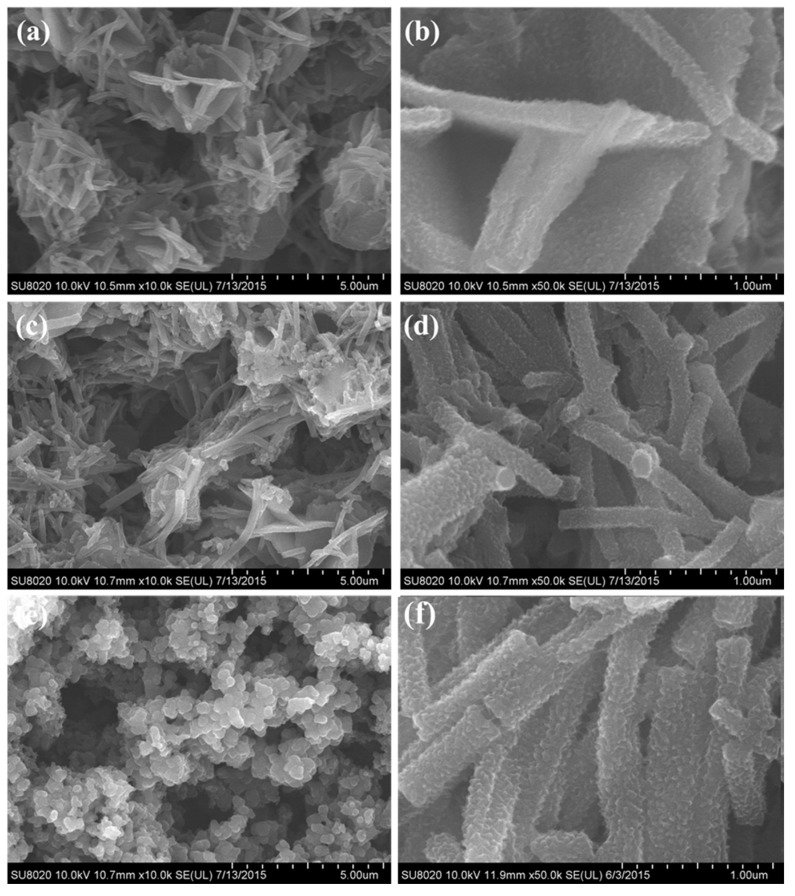
SEM images of the PANI samples synthesized with CA/Ani = (**a**) 0.01, (**b**,**c**) 0.1, (**d**) 0.5, (**e**) 2, and (**f**) the morphologies of the PANI at 4 h. The others reaction conditions: APS/Ani = 1, in ice bath for 24 h.

To investigate the effects of CA on the final morphology of the products, we further changed the values of CA/Ani and took the reaction under the same conditions. Figure 5 shows the SEM images of the samples that were synthesized at CA/Ani = 0.01, 0.1 and 2. When compared with Figure 3 (CA/Ani = 0.5), we can see that the morphologies of the final samples were greatly affected by the values of CA/Ani. When the CA/Ani value is low (like 0.01), nanoflowers stacked by nanosheets with the thickness about 150 nm were obtained (Figure 5a). When the CA/Ani value increased to 0.1, the products contained nanotubes and nanosheets, and the average diameter of the nanotubes was about 194 nm (Figure 5b,c). When the CA/Ani value came to 0.5, uniform nanotubes were attained, as discussed above (Figure 5d). But when further increasing the CA/Ani value to 2, reunited nanoparticles with average diameter about 297 nm were obtained (Figure 5e). In order to obtain the morphologies of the PANI in the beginning, the morphologies of the PANI at 4 h were shown in Figure 5f. It can be seen that the surfaces of PANI nanotubes was also rough, and a large number of nanotubes were generated in the beginning. However, in order to make the reaction more complete, the reaction time was prolonged to 24 h. The morphologies of the products were determined in the beginning, and it was almost the same when polymerization was further prolonged. The information was summarized in Table 1.

Why the CA/Ani values influenced the final morphology so much? According to previous reports, when CA was used as dopant, the micelle formed by the citric acids and aniline monomer acts as a soft template and the hydrogen bonding provides the driving force in the self-assembly process of nanostructures [44]. CA reacts with aniline monomer to form the PANI salt that shows poor solubility. In the aqueous solution, the salt is surrounded by free CA and Ani to form the micelle, and the micelle has the core-shell structure, which the inner core exerts the hydrophobic nature, which was composed with CA/Ani salts, free CA, and Ani serves as the outer shell of the micelle. So the relative amount of CA and Ani affects the composition and the amount of the micelle. When at a low CA/Ani value, the amount of CA is far less than Ani, and thus results in less micelle in the system, and the micelle is composed with large amounts of Ani monomer and the salts. While increasing the molar ratio, the amount of the as formed micelle increases, and the micelle is mainly composed with CA and the salts. When the concentration of the micelle achieves the saturation, the micelle will precipitate. It is believed that the composition and the size of the micelle determine the morphologies of PANI nanostructures. Since the oxidation APS is hydrophilic, the polymerization reaction only occurs at the water/micelle interface [45]. The growth of the nanostructures is controlled by the accretion and elongation process [46]. With the polymerization proceeding, the size of the micelle would become larger by the accretion process to form nanosheets at the low micelle concentration, and finally stack to three dimensional nanoflows. In the polymerization proceeding, the N in polyaniline molecule is the donor of hydrogen bond, and there are a large number of hydrogen bond receptors in the molecular structure of citric acid. The interaction between them plays the role of hydrogen bond induction in the synthesis of PANI nanotubes [26,27]. The micelle would grow to nanotubes in the direction of the hydrogen bonds by the elongation process at the appropriate micelle concentration. While the micelle concentration is too high, the growth of the micelles tends to aggregate and finally formed the nanoparticles. The secondary growth of PANI on the surface of the as formed PANI nanostructures makes the surface scratchy and it was easily seen in the SEM images.

**Figure 6:**
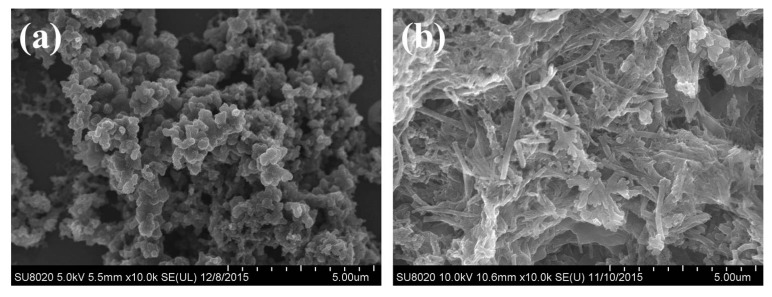
SEM images of the samples prepared at CA/Ani = 0.5, APS/Ani = 1, (**a**) at room temperature for 24 h; (**b**) at ice bath with mechanical stirring for 24 h.

Depending on the above discussion, we can see the hydrogen bonding plays a vital role in the formation of PANI nanotubes. To investigate the effect of the hydrogen bonding, we perform another two experiments that destroy the hydrogen bonding in the reaction solution by raising the reaction temperature and adapting mechanical stirring. Figure 6a shows the reaction at 25 °C with the molar amount of APS/Ani = 1, CA/Ani = 0.5 and no mechanical stirring, which was compared with Figure 3. As can be seen, when the polymerization was at a higher reaction temperature, nanoparticles were obtained, which were aggregated with each other rather than forming uniform nanotubes. Figure 6b shows the effect of mechanical stirring on the morphology, with the molar amount of APS/Ani = 1, CA/Ani = 0.5 in the ice bath for 24 h. A small quantity of nanotubes can be observed, most of the products were irregular agglomerated. It is well known that a high reaction temperature and mechanical stirring can destroy the hydrogen bonding in the solution [47], the hydrogen bonding between the polymers [48] plays the decisive role in the formation of uniform nanostructures. So when the hydrogen bonding was destroyed, no regular nanotubes were obtained. 

### 3.3. Gas Sensing Properties

Based on the as synthesized PANI nanomaterials with different morphologies doped with citric acid, we fabricated the nanomaterials into the sensors as described in the experiments section. Then the sensing performances of the sensors to NH_3_ were measured in ambient air at room temperature.

**Figure 7:**
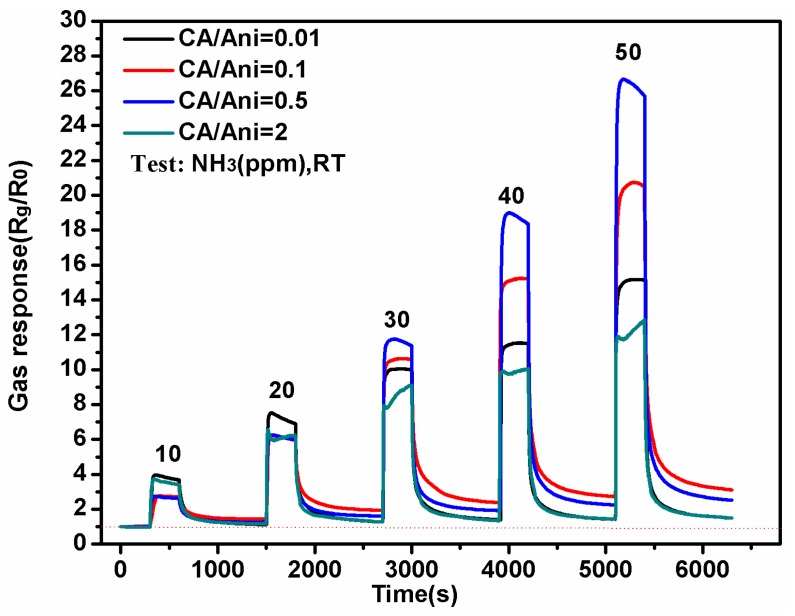
Gas sensing response of the sensors deposited with PANI nanomaterials with different morphologies to ammonia ranging from 10 to 50 ppm at room temperature.

**Figure 8:**
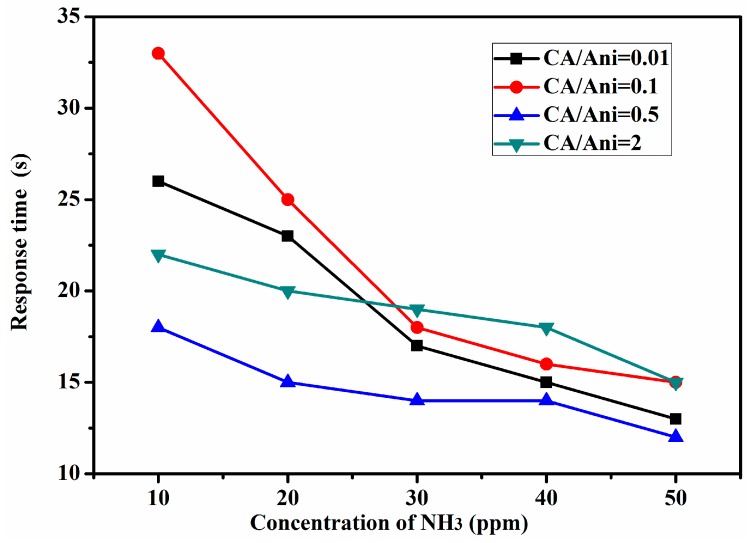
Response time the sensors deposited with PANI nanomaterials with different morphologies to ammonia ranging from 10 to 50 ppm at room temperature.

Figure 7 shows the sensing response of the sensors with different morphologies to NH_3_, ranging from 10 ppm to 50 ppm at room temperature. The gas response of the sensors is defined as R_g_/R_0_, in which R_g_ and R_0_ represent the resistances of the sensor devices after and before exposure to NH_3_, respectively. Figure 8 shows the response time of the sensors with different morphologies to NH_3_ ranging from 10 ppm to 50 ppm at room temperature. The response time is defined as the time for reaching 90% of the full response change of the sensor after the testing gas gets in. From Figure 7, we can see that when NH_3_ is injected into the test chamber, the resistance of the sensing devices increased immediately. The polyaniline nanoflowers synthesized at CA/Ani = 0.01 shows better response to NH_3_ of 10 and 20 ppm, while with the increase of NH_3_ concentration, the response behaves nonlinear decline. The response of the devices of nanotubes obtained at the molar ratio of CA/Ani = 0.5 shows well the linear increase relationship to the increase of NH_3_. The response of polyaniline nanotubes sensor to 50 ppm NH_3_ is as high as about 28. In general, the sensors based on the uniform PANI nanotubes show higher sensing sensitivity to NH_3_ when comparing to sensors with other morphologies. From Figure 8, we can see that with the increasing of NH_3_, the response time decreased, and the sensor based on the PANI nanotubes exhibits a shorter response time. Particularly, when further increasing the CA/Ani value to 2, the lager reunited nanoparticles with average diameter about 297 nm were obtained, which was quite different from other sample (nanosheets and nanotubes), as shown in the Figure 5. This kind of structure was similar to bulk powder material. The working mechanism of a gas sensor depends on the convection of electrical conductivity for surface reactions (such as oxidation or reduction) caused by different gas exposures [49]. These surface reactions rely on the defects and active centers on the surface layer of materials, the sensor response time is usually determined by the surface-to-volume ration of material [49]. When compared with bulk powder material, nanosheets and nanotubes have more excellent gas sensing properties due to the high surface-to-volume ration and large surface activities [50]. So under higher gas concentrations, they may take less time to reach an equilibrium position. When concentration. of NH_3_ was 30 ppm for CA/Ani = 2, the response time was obviously prolonged when compared to the other samples. Preliminarily, we can conclude the PANI nanotubes sensor shows better response to NH_3_.

**Figure 9:**
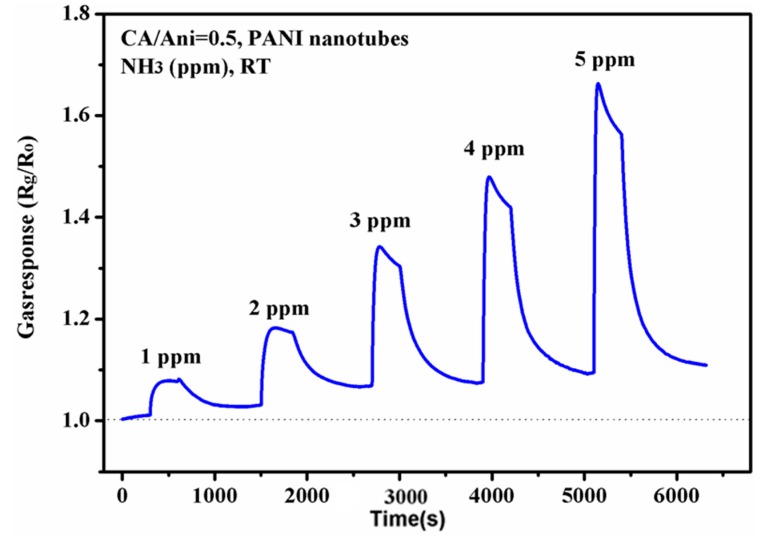
Gas sensing response of the nanotubes sensor to ammonia ranging from 1 to 5 ppm at room temperature.

Then, we further measured the sensing response of the nanotubes sensor to NH_3_ with a lower concentration ranging from 1 to 5 ppm at room temperature, which is shown in Figure 9, and the gas response is defined as above. The sensitivity to 1 ppm NH_3_ is about 1.1, and with the increasing concentration of NH_3_ to 5 ppm the sensor shows a well linear increase. It is also found that the baseline lifted up slowly as the test processing. Overall, we can conclude that the polyaniline nanotubes sensor has a good response to NH_3_.

The sensing mechanism of the polyaniline nanotubes to NH_3_ is thought the chemical absorption of NH_3_ to change the conductivity of the polyaniline chain [47]. By the doping process, the H^+^ of the citric acid combines the N of the imine in the polymer chain resulting in protonation, and thus the conductivity is increased [19]. When contacting with NH_3_, the NH_3_ molecular will preferentially seize the H^+^ between the polymer chains to form ammonium ion, resulting in the decrease of conductivity. The reaction is reversible. When NH_3_ is taken away, the conductivity would recover. From the discussions above, we can see that the tubular polyaniline shows a better response to NH_3_ than the other samples. As discussed before, tubular polyaniline has abundant polarons, which act as sensing-sensitivity sites on the PANI chains that improved the sensitivity.

**Figure 10:**
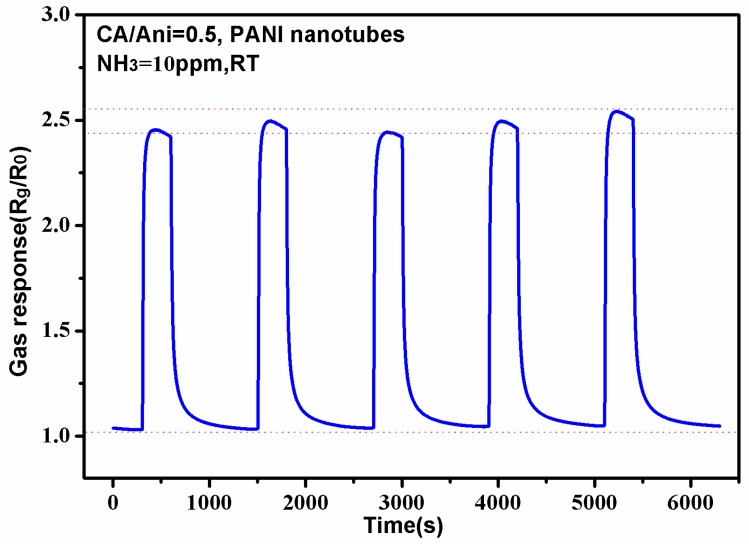
Sensing response of the nanotubes sensor to ammonia of 10 ppm at room temperature.

Depending on the discussion above, we can conclude preliminarily that the PANI nanotubes sensors show a better response to NH_3_ than ones with other morphologies, thus we only test the stability and selectivity of the sensors based on PANI nanotubes. Figure 10 shows the gas response to NH_3_ of 10 ppm with five repeats at room temperature of the PANI nanotubes sensor. From the picture, we can see that the sensing sensitivities to NH_3_ of 10 ppm are all about 2.5, which match well with the response in Figure 7.

**Figure 11:**
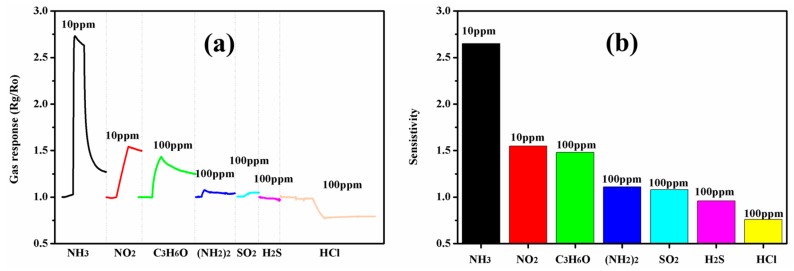
The sensing responses of the sensor based on the PANI nanotubes to NH_3_ (10 ppm), NO_2_ (10 ppm), C_3_H_6_O (100 ppm), NH_2_NH_2_ (100 ppm), SO_2_ (100 ppm), H_2_S (100 ppm), and HCl (100 ppm). (**a**) The real time patterns; (**b**) gas responses contracts.

The selectivity of MOS gas sensors is of great importance. Here, nitrogen dioxide (NO_2_), acetone (C_3_H_6_O), hydrazine (NH_2_NH_2_), sulfur dioxide (SO_2_), hydrogen sulfide (H_2_S), and hydrogen chloride (HCl) were chosen as the interfering gases. The real time response and sensitivity contrast histogram were exhibited in Figure 11. According to the special sensing mechanism, H_2_S and HCl enhance the protonation effect, and thus exhibits the opposite response when compared to other gases. The gas response to NO_2_ of 10 ppm and C_3_H_6_O of 100 ppm are 1.52 and 1.48, respectively, which are obviously lower than the gas response to NH_3_ of 10 ppm. The sensing performance to NO_2_ and acetone is quite different to ammonia, as can be seen in Figure 11a, the response time of the sensor to NO_2_ and acetone is much longer than to ammonia. So we can conclude that the sensor based on PANI nanotubes shows good selectivity to NH_3_.

**Figure 12:**
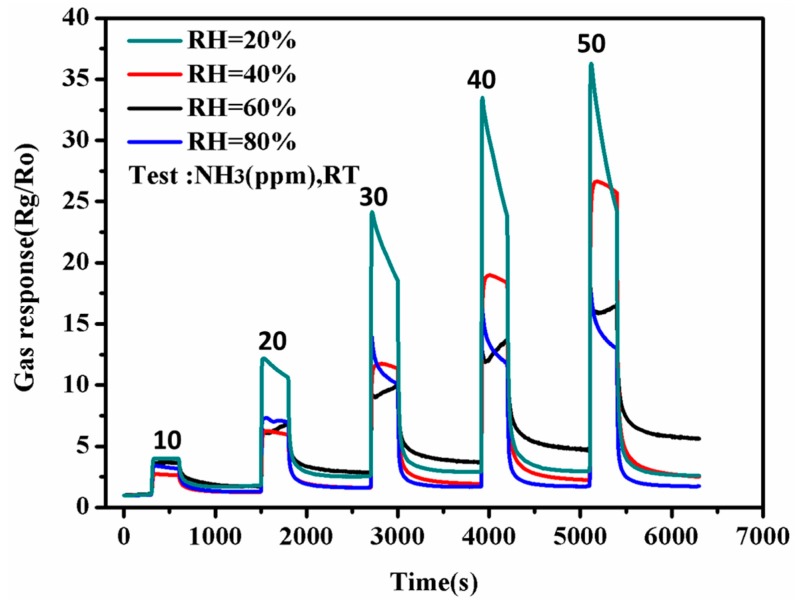
The influence of relative humidity on the performances of nanotubes sensor to ammonia from 10 to 50 ppm at room temperature (between 20% and 80% of relative humidity (RH)).

As the sensor performances is greatly influenced by the value of relative humidity (RH), and all of the above experimental results were obtained with 40% of RH at room temperature. Now the value of RH was changed between from 20% to 80%, and the results were shown in Figure 12. It can be seen that as the value of RH increased, the performance of nanotubes sensor to ammonia became worse from 10 to 50 ppm. At 80% of RH, it had the worst performance. The reason is that as the value of RH increased, the detection environment contained more water vapor. The active site of PANI nanotubes was occupied by more water vapor, which used to occupy and interact to NH_3_. This resulted in the decrease of detection site to NH_3_, and the performances of PANI nanotubes sensor became worse.

## 4. Conclusions

In summary, using the citric acid as the dopant, by optimizing the synthesis conditions we had synthesized high quality polyaniline nanotubes with uniform morphology and size, and the effects of synthesis conditions to the morphology of the polyaniline nanotubes were explored. Further, we studied the gas sensing properties of the polyaniline nanotubes and compared its gas sensing properties with the other micro/nanostructures. The results show that the polyaniline nanotubes sensor exhibits better sensing response, good stability, and selectivity.

## Figures and Tables

**Table 1:** Effect of molar ratio of CA/Ani on the morphology of the products with the synthesis conditions: ice bath, certain amount of Ani and APS.

CA/Ani Value	Morphology	Size
0.01	Nanoflowers (nanosheets)	150 nm
0.05	Nanosheets and nanotubes	194 nm
0.5	Nanotubes	210 nm
2	Nanoparticles	297 nm

## References

[1] Jia L., Cai W.P. (2010). Micro/Nanostructured Ordered Porous Films and Their Structurally Induced Control of the Gas Sensing Performances. Adv. Funct. Mater..

[2] Sira S., Tapan S., Raul H., Ashok M. (2017). Potassium Iodide-Functionalized Polyaniline Nanothin Film Chemiresistor for Ultrasensitive Ozone Gas Sensing. Polymers.

[3] Su X.S., Gao L., Zhou F., Duan G.T. (2017). A substrate-independent fabrication of hollow sphere arrays via template-assisted hydrothermal approach and their application in gas sensing. Sens. Actuators B Chem..

[4] Su X.S., Gao L., Zhou F., Cai W.P., Duan G.T. (2017). “Close network” effect of a ZnO micro/nanoporous array allows high UV-irradiated NO_2_ sensing performance. RSC Adv..

[5] Zhu Y.D., Wang Y.Y., Duan G.T., Cai W.P. (2015). In situ growth of porous ZnO nanosheet-built network film as high-performance gas sensor. Sens. Actuators B Chem..

[6] Dai Z.F., Xu L., Duan G.T., Li T., Zhang H.W., Li Y., Wang Y., Wang Y.L., Cai W.P. (2013). Fast-Response, Sensitivitive and Low-Powered Chemosensors by Fusing Nanostructured Porous Thin Film and IDEs-Microheater Chip. Sci. Rep..

[7] Dai Z.F., Duan G.T., Cheng Z.X., Xu L., Li T., Liu G.Q., Zhang H.W., Li Y., Cai W.P. (2015). Janus gas: Reversible redox transition of Sarin enables its selective detection by an ethanol modified nanoporous SnO_2_ chemiresistor. Chem. Commun..

[8] Byshkin M., Buonocore F., Matteo A.D., Milano G. (2015). A unified bottom up multiscale strategy to model gas sensors based on conductive polymers. Sens. Actuators B Chem..

[9] Seon J.P., Chul S.P., Hyeonseok Y. (2017). Chemo-Electrical Gas Sensors Based on Conducting Polymer Hybrids. Polymers.

[10] Nicolas D.D., Poncin-Epaillard F. (2003). Polyaniline as a new sensitive layer for gas sensors. Anal. Chim. Acta.

[11] Cho S., Kwon O.S., You S.A., Jang J. (2013). Shape-controlled polyaniline chemiresistors for high-performance DMMP sensors: Effect of morphologies and charge-transport properties. J. Mater. Chem. A.

[12] Virji S., Huang J.X., Kaner R.B., Weille B.H. (2004). Polyaniline nanofiber gas sensors: Examination of response mechanisms. Nano Lett..

[13] Huang J.X., Kaner R.B. (2004). A general chemical route to polyaniline nanofibers. J. Am. Chem. Soc..

[14] Virji S., Fowler J.D., Baker C.O., Huang J.X., Kaner R.B., Weille B.H. (2005). Polyaniline nanofiber composites with metal salts: Chemical sensors for hydrogen sulfide. Small.

[15] Thanh H.L., Yukyung K., Hyeonseok Y. (2017). Electrical and Electrochemical Properties of Conducting Polymers. Polymers.

[16] Paik P., Manda R., Amgoth C., Kumar K.S. (2014). Polyaniline nanotubes with rectangular-hollow-core and its self-assembled surface decoration: High conductivity and dielectric properties. RSC Adv..

[17] Li Z.F., Zhang H.Y., Liu Q., Liu Y.D., Stanciu L., Xie J. (2014). Covalently-grafted polyaniline on graphene oxide sheets for high performance electrochemical supercapacitors. Carbon.

[18] Poldsalu I., Harjo M., Tamm T., Uibu M., Peikolainen A.L., Kiefer R. (2017). Inkjet-printed hybrid conducting polymer-activated carbon aerogel linear actuators driven in an organic electrolyte. Sens. Actuators B Chem..

[19] Zhang H.D., Tang C.C., Long Y.Z., Zhang J.C., Huang R., Li J.J., Gu C.Z. (2014). High-sensitivity gas sensors based on arranged polyaniline/PMMA composite fibers. Sens. Actuators A Phys..

[20] Park H.W., Kim T.Y., Huh J., Kang M., Lee J.E., Yoon H. (2012). Anisotropic Growth Control of Polyaniline Nanostructures and Their Morphology-Dependent Electrochemical Characteristics. ACS Nano.

[21] Wan M. (2009). Some Issues Related to Polyaniline Micro-/Nanostructures. Macrom. Rap. Commun..

[22] Wang F., Wang Z.J., Tana M.H., He C.B. (2015). Uniform Polyaniline Nanotubes Formation via Frozen Polymerization and Application for Oxygen Reduction Reactions. Macrom. Chem. Phys..

[23] Rana U., Chakrabarti K., Malik S. (2012). Benzene tetracarboxylic acid doped polyaniline nanostructures: Morphological, spectroscopic and electrical characterization. J. Mater. Chem..

[24] Bhattacharya S., Rana U., Malik S. (2013). Relaxation Dynamics and Morphology-Dependent Charge Transport in Benzene-Tetracarboxylic-Acid-Doped Polyaniline Nanostructures. J. Phys. Chem. C.

[25] Tavandashti N.P., Ghorbani M., Shojaei A. (2013). Controlled growth of hollow polyaniline structures: From nanotubes to microspheres. Polymer.

[26] Wu W.L., Pan D., Li Y.F., Zhao G.H., Jing L.Y., Chen S.L. (2015). Facile fabrication of polyaniline nanotubes using the self-assembly behavior based on the hydrogen bonding: A mechanistic study and application in high-performance electrochemical supercapacitor electrode. Electrochim. Acta.

[27] Zhang L.J., Long Y.Z., Chen Z.J., Wan M.X. (2004). The effect of hydrogen bonding on self-assembled polyaniline nanostructures. Adv. Funct. Mater..

[28] Yin C.Q., Duan G.T., Cai W.P. (2016). Polyaniline nanofibers and their self-assembly into a film to be used as ammonia sensor. RSC Adv..

[29] Mu J.J., Ma G.F., Peng H., Li J.J., Sun K.J., Lei Z.Q. (2013). Facile fabrication of self-assembled polyaniline nanotubes doped with d-tartaric acid for high-performance supercapacitors. J. Power Sources.

[30] Xu G.H., Xu D.D., Zhang J.N., Wang K.X., Chen Z.M., Chen J.F., Xu Q. (2013). Controlled fabrication of PANI/CNF hybrid films: Molecular interaction induced various micromorphologies and electrochemical properties. J. Colloid Interface Sci..

[31] Xu C., Chen H., Jiang F. (2015). Adsorption of perflourooctane sulfonate (PFOS) and perfluorooctanoate (PFOA) on polyaniline nanotubes. Colloid Surf. A Phys. Eng. Asp..

[32] Zhang L.J., Wan M.X. (2003). Self-assembly of polyaniline—From nanotubes to hollow microspheres. Adv. Funct. Mater..

[33] Rana U., Mondal S., Sannigrahi J., Sukul P.K., Asif A.M., Majumdar S., Malik S. (2014). Aromatic bi-, tri- and tetracarboxylic acid doped polyaniline nanotubes: Effect on morphologies and electrical transport. J. Mater. Chem. C.

[34] Chiou N.R., Lee L.J., Epstein A.J. (2007). Self-assembled polyaniline nanofibers/nanotubes. Chem. Mater..

[35] Sim B., Choi H.J. (2015). Facile synthesis of polyaniline nanotubes and their enhanced stimuli-response under electric fields. RSC Adv..

[36] Ding H.J., Shen J.Y., Wan M.X., Chen Z.J. (2008). Formation Mechanism of Polyaniline Nanotubes by a Simplified Template-Free Method. Macrom. Chem. Phys..

[37] Huang K., Zhang Y.J., Long Y.Z., Yuan J.H., Han D.X., Wang Z.J., Niu L., Chen Z.J. (2006). Preparation of highly conductive, self-assembled gold/polyaniline nanocables and polyaniline nanotubes. Chem. A Eur. J..

[38] Long Y.Z., Long Y.Z., Zhang L.J., Ma Y.J., Chen Z.J., Wang N.L., Zhang Z., Wan M.X. (2003). Electrical conductivity of an individual polyaniline nanotube synthesized by a self-assembly method. Macromol. Rapid Commun..

[39] Kosonen H., Ruokolainen J., Knaapila M., Torkkeli M., Jokela K., Serimaa R., Brinke G.T., Bras W., Monkman A.P., Ikkala O. (2000). Nanoscale conducting cylinders based on self-organization of hydrogen-bonded polyaniline supramolecules. Macromolecules.

[40] Qiu H.J., Wan M.X., Matthews B.R., Dai L.M. (2001). Conducting polyaniline nanotubes by template-free polymerization. Macromolecules.

[41] Stejskal J., Sapurina I., Trchova M., Konyushenko E.N. (2008). Oxidation of aniline: Polyaniline granules, nanotubes, and oligoaniline microspheres. Macromolecules.

[42] Zujovic Z.D., Laslau C., Bowmaker G.A., Kilmartin P.A., Webber A.L., Brown S.P., Travassejdic J. (2010). Role of Aniline Oligomeric Nanosheets in the Formation of Polyaniline Nanotubes. Macromolecules.

[43] Ran F., Tan Y.T., Liu J., Zhao L., Kong L.B., Luo Y.C., Kang L. (2012). Preparation of hierarchical polyaniline nanotubes based on self-assembly and its electrochemical capacitance. Polym. Adv. Technol..

[44] Tavandashti N.P., Ghorbani M., Shojaei A. (2015). Morphology transition control of polyaniline from nanotubes to nanospheres in a soft template method. Polym. Int..

[45] Yang Y., Chen S., Xu L. (2011). Enhanced conductivity of polyaniline by conjugated crosslinking. Macromol. Rapid Commun..

[46] Collie Duguid E.S., Sweeney K., Stewart K.N., Miller I.D., Smyth E., Heys S.D. (2012). SerpinB3, a new prognostic tool in breast cancer patients treated with neoadjuvant chemotherapy. Breast Cancer Res. Treat..

[47] Li D., Huang J., Kaner R.B. (2009). Polyaniline Nanofibers: A Unique Polymer Nanostructure for Versatile Applications. Acc. Chem. Res..

[48] Long Y., Chen Z.J., Wang N.L., Ma Y.J., Zhang Z., Zhang L.J., Wan M.X. (2003). Electrical conductivity of a single conducting polyaniline nanotube. Appl. Phys. Lett..

[49] Ponzoni A., Comini E., Sberveglieri G., Zhou J., Deng S.Z., Xu N.S., Ding Y., Wang Z.L. (2006). Ultrasensitive and highly selective gas sensors using three-dimensional tungsten oxide nanowire networks. Appl. Phys. Lett..

[50] Zhang J., Liu X.H., Neri G., Pinna N. (2016). Nanostructured materials for room-temperature gas sensors. Adv. Mater..

